# Chemical characterization of carbonaceous carbon from industrial and semi urban site of eastern India

**DOI:** 10.1186/s40064-016-2506-9

**Published:** 2016-06-22

**Authors:** Basant Shubhankar, Balram Ambade

**Affiliations:** Department of Chemistry, National Institute of Technology, Jamshedpur, Jharkhand 831014 India

**Keywords:** PM_10_, EC, OC, WSOC, Eastern India

## Abstract

Rigorous campaign was carried out from July 2013 to June 2014 at the remote and industrial site (Adityapur and Seraikela Kharsawan) in the eastern India aiming to identify and quantify the changes of aerosol chemical composition in the presence of industrial and biomass burning influence. The 24-h PM_10_ filter samples were analyzed by mass, carbonaceous species, organic ions. The results suggested that the average PM_10_ concentrations were 165 ± 43.93, 141 ± 30.86 μg/m^3^ in industrial and remote site respectively. Secondary organic ions (SOC) were the dominant pollutants of PM_10_. Total carbon was a significant component explaining above 15 % of PM_10_. The annual average mass concentration of EC, OC, WSOC 26.39 ± 4.56, 5.11 ± 1.82, 18.56 ± 5.30 and 16.27 ± 5.75, 7.70 ± 2.1, 9.65 ± 1.92 µg/m^3^, OC/EC, WSOC/OC 5.29 ± 1.08, 0.71 ± 0.17 and 2.34 ± 0.75, 0.67 ± 0.16) of industrial and remote site were respectively; and OC/EC particularly in industrial site it reached the highest 5.29 ± 1.08 which demonstrated that SOC should be a significant composition of PM_10_. The mass fraction of the highlighted species varies seasonally, resulting the air mass trajectories and corresponding cause severe strength. Based on exact mass concentration ratios of EC/OC, WSOC/OC, we predicted that industries and biofuel/biomass burning are a major source of atmospheric aerosols in the eastern part of India. This study provides the scientific baseline data of carbonaceous aerosols for eastern Jharkhand, India.

## Background

Carbonaceous aerosols (CA) in environments have received special attention recently in the modern India, because of their effects on the local environment, water resources, agriculture production, ambient air quality, decline, visibility and public health (Jacobson 2001). The CA includes two components, organic carbon (OC) and elemental carbon (EC) also known to be as black carbon (BC), which constitutes a major fraction of PM (Putaud et al. 2004). All these CA is one of the most important and is ubiquitous materials found in the atmosphere, formed by all types of combustion processes (industrial, biomass burning, etc.). EC is essentially considered as a primary pollutant which is directly emitted by the incomplete combustion of fossil fuel and biomass burning (Seinfeld and Pandis 1998). It has also been noted that the biomass burning emission are one of the prime sources of EC, which is often used as a marker for the pollution (Ambade 2015); furthermore, its temporal pattern could be connected to traffic concentration (Ruellan and Cachier 2001). The EC is an essential constituent in atmospheric aerosols and is typically considered as the only particulate-phase light-absorbing species (also known as brown carbon) in the earth’s radiation budget (Jacobson 2001). Similarly OC has both primary and secondary origin. Primary OC is mostly formed during incomplete combustion processes such as unleaded gasoline burning in an atmosphere or field agricultural and biomass burning (Cachier et al. 1991; Duan et al. 2004). It is also directly discharged from plant spores, pollens and dirt organic matter. Secondary organic carbon (SOC) can originate from different processes such as gas to particle translation of low vapor pressure volatile organic compounds (VOCs), condensation, physical and chemical adsorption beside these aqueous phase processes are also important to generate SOA and water soluble organics (Wonaschütz et al. 2011; Duong et al. 2011). The presence of SOA is recommended by an increase of the OC/EC and WSOC/OC ratio. SOA can be easily estimated using EC as a tracer of OC primary emission (Salma et al. 2004).

Both CA i.e. (OC and EC) in particulate matter (PM) play very crucial roles in visibility degradation and climate effects (IPCC 2001). The EC, which is often related with light-absorbing and optically-derived, black carbon (BC), which is known to cause heating in the air on a local scale, thus changing the atmospheric constancy and vertical mixing, and distressing large-scale circulation of air and also the hydrological cycle (Menon et al. 2002). Water soluble organic compounds (WSOCs) represent a considerable segment of atmospheric organic matter, accounting for 10–90 % of OC content in ambient aerosols depending on locations (Pöschl 2005). In ambient aerosols, it consists of a large variety of chemical species: a hydrosugars, alcohols, sugars, aliphatic and aromatic acids, amino acids and aliphatic amines, etc., as well as large and medium size, convoluted molecules such as Humic like Substances (HULIS) (Graber and Rudich 2006). WSOC plays an important role in global climate change by changing the hygroscopicity of atmospheric aerosols (Wonaschütz et al. 2013). Besides biomass burning emissions, the WSOC has contributions from SOA in the atmosphere, occurring through photochemical reactions of VOC. The association of these WSOC has been good, predictable in an influencing the number density of cloud condensation nuclei (CCN) (Crosbie et al. 2015) and shifting the radiation balance of the atmosphere (Kaiser et al. 2011). WSOC can also cause a deleterious consequence on human health by enhancing the solubility of toxic pollutants (Kondo et al. 2007). Moreover, some of WSOCs are allergens, leading to respiratory and other related diseases (Franze et al. 2005).

The aim and objective of the present study is to provide most novel and a better understanding of the characteristics of the aerosol particles for carbonaceous species in industrial (Adityapur) and remote (Seraikela Kharsawan) atmosphere. For this purpose OC, EC and WSOC associated to PM_10_ fraction were investigated rigorously for 1 year. In addition above, this study also magnify contributions of OC and EC at both sites of industries process and its emission, coal combustion, vehicular exhaust and biomass burring etc. Besides all these, we are also presenting for the first time the upshots of the air mass trajectories and AOD involved during this 1 year sampling of the two sites.

## Experimental details

### Site description

Adityapur (22°78′19″N, 86°15′19″E) is one of the largest industrial areas in eastern India and a Special Economic Zone (SEZ) is in the works through the Adityapur Industrial Development Authority (AIDA). The Adityapur Industrial Estate which is in the area of 33,970 acres, 53 sq. mile has been Asia’s largest industrial hub. About 1000 medium and small scale industries are located here and about 250 are under the process of construction. There are about 20 large scale industries such as TGS, Usha Martin, Adhunik Group, and RSB group of industries are situated in the Adityapur.

**Fig. 1:**
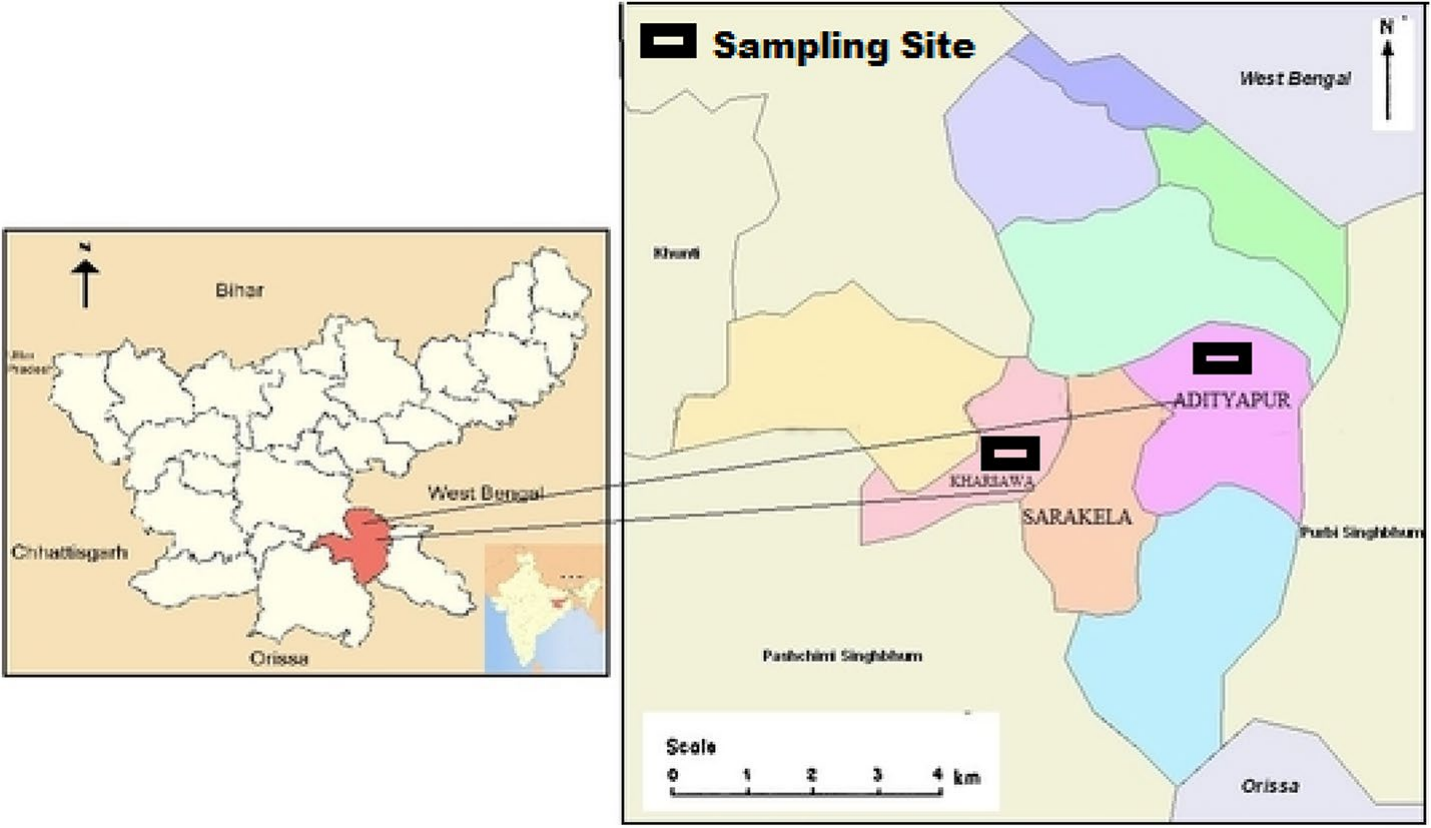
Map of study area with sampling site

Seraikela-Kharsawan (22°29′26″N, 85°30′14″E) which has the remote/rural background, is at a distance of 57 km from the Adityapur. The main terrain of Seraikela-Kharsawan is Chhotanagpur plateau. It is situated in a Dalma mountain region, which is covered with a dense belt of forests (Census 2011). According to the govt of India census record of 2011 Seraikela Kharsawan district has a population of 1,063,458. The sampling site has been shown in Fig. 1.

#### Meteorology

**Fig. 2:**
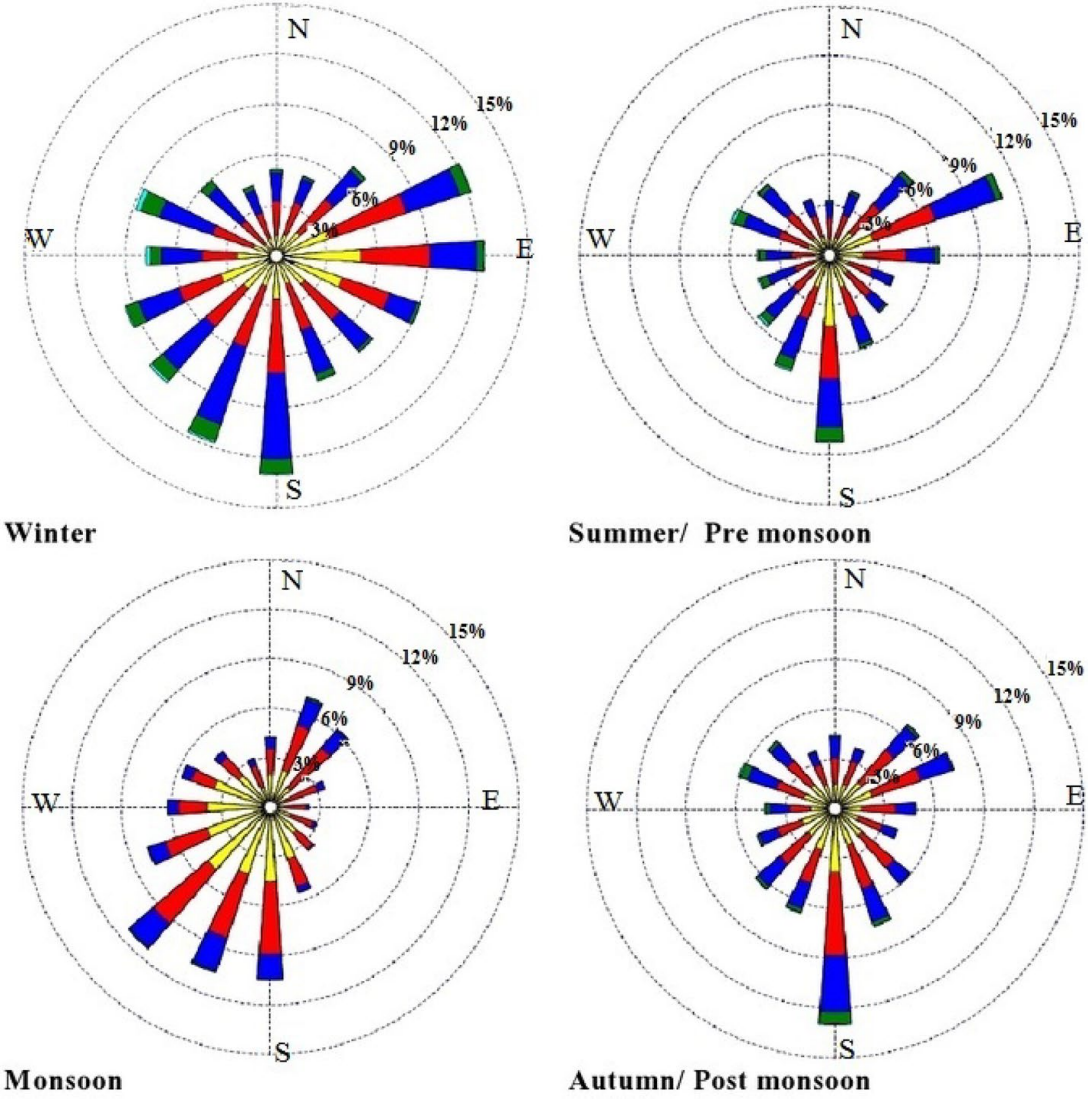
Seasonal wind rose plot of sampling site

According to the meteorological station of Adityapur and Saraikela Kharsawan (both sampling sites), the ambient temperature and relative humidity were varied from 8.0 to 41.0 °C (avg. 24.5 °C) and 22 to 95 % (avg. 61 %) in winter respectively, whereas in summer their ranges were 19.0–45.0 °C (avg. 30 °C) and 31–81 % (avg. 60 %), respectively. The details of meteorological data during the sampling period were presented in (Table 1). It should be also noted that during rainfall days no sampling was taken. Due to a robust land/sea thermal gradient, a clear diurnal oscillation was perceived in wind speed and direction (Pavuluri et al. 2010) but as it has been seen that the wind contrast decreases with gaining height and finally disappears above 1 km (Ambade 2014). The wind directions with wind speed are summarized in Fig. 2.

**Table 1:** The ranges, average and for meteorological parameters at sampling sites during 1 July 2013–30 June 2014

Month	Temperature (°C) Average (max–min)	Pressure (hpa) Average (max–min)	Precipitation (mm) Average (max–min)	Humidity (%) Average (max–min)
Winter	24.5 (41.0–8.0)	1014 (1023–1002	4.0 (17.0–0.0)	61 (22–95)
Summer/pre monsoon	30.0 (45.0–19.0)	1000 (1026–991)	52.0 (108.0–0.0)	60 (31–81)
Monsoon	29.0 (26.0–23.0)	1000 (1009–991)	7.3 (108.0–0.0)	87 (75–96)
Post monsoon/autumn	16.0 (33.0–27.0)	1016 (1019–997)	0.27 (33.0–0.0)	71 (26–95)

### PM_10_ sampling and mass measurement

PM_10_ (<10 μm aerodynamic diameter) aerosol samples (N = 52) was collected from each sampling site of Seraikela-Kharsawan and Adityapur during 1 July, 2013–30 June, 2014. The sampling details and standard operational parameter of sampling equipment used at two sites are presented in Table 2.

**Table 2:** An overview of the both sampling campaign includes the sampling equipment used, number of filter with size etc

Sampling site (with code)	Seraikela-Kharsawan	Adityapur
Site category	Semi urban	Industrial
Date start	01-07-2013	03-07-2013
Date stop	30-06-2014	02-06-2014
Number of days	52	52
Number of valid samples	50	50
% of valid samples	96 %	94 %
Number of OC/EC data	10	10
Number of WSOC data	40	40
No of field blank	12	12
No of lab blank	12	11
Aerosol sampler	RDS-460 Envirotech make, CSIR-NEERI improved Technology, India	RDS-460 Envirotech make, CSIR-NEERI improved Technology, India
Filter size (d) (mm)	47	47
Flow rate (L min^−1^)	3	3

Aerosol samples were collected on 47 mm high purity PALLFLEX^®^™ tissue quartz filters (pre-combusted at 900 °C for 3 h to remove organic artifacts or impurities) using a respirable dust sampler (Envirotech RDS 460, CSIR NEERI improved Technology, India). The flow rate of the sampler was periodically calibrated and was about ~3 L min^−1^. After collection, filters were stored in refrigerator at ∼4 °C prior to chemical analysis to prevent the loss of volatile components. Furthermore the 5 % of field blanks were collected to subtract the positive artifacts that resulted from adsorption of gas-phase organic compounds onto the filter during and/or after sampling. Negative artifacts due to the volatilization of particle-phase organics from particle samples were not quantified in this study. The particulate mass concentrations (PM_10_) were obtained gravimetrically from initial and final weight of the filters. Thereafter the Loaded and unloaded filters were conditioned for constant relative humidity (RH) of 45 ± 5 % and temperature 22 ± 2 % for 48 h calibration before weighing in an analytical balance (Metler M×5 microbalance; Metler Toledo Co. Inc. Greifensee, Switzerland) with ±1 mg sensitivity.

Furthermore the sample filters were subsequently analyzed for carbonaceous species (EC, OC and WSOC).

### Analysis of EC, OC

In the present study, we measured EC and OC on sunset EC-OC analyzer using NIOSH (National Institute for Occupational Safety and Health) protocol (Rengarajan et al. 2011). In addition to this, procedural filter blanks were analyzed (N = 12) and mass concentrations were suitably corrected for blanks. An external standard named as Potassium hydrogen phthalate (KHP) is used as to validate the precision of the measurement of OC during the analysis process and an overall analytical uncertainty of not more than 4 % were given to the given analysis.

### Analysis of WSOC

The total organic carbon (TOC) analyzer (Shimadzu, model TOC-5000A) were used for the measurement of the concentration of water-soluble organic carbon (WSOC) (Ram et al. 2010a). For the determination of WSOC include sonication of 1–2 strokes (3.14 cm^2^ each) of sample with 30/40 mL Milli-Q water for estimated 30 min, followed by extracting the filtrate using a glass-syringe while passing through a glass-fiber filter (25 mm diameter) into a pre-cleaned amber coloured glass vials, and subsequent analysis on a TOC analyzer.

### Air-mass back trajectories

In order to identify sources and to examine how pollutant transport paths affect concentrations of air pollutants at the sampling site, a 4-day backward trajectory analysis is performed for each air-mass case. The analysis was calculated with the assist of the HYSPLIT (Hybrid Single-Particle Lagrangian-Integrated Trajectory) model (Draxler and Rolph 2003). In our present study the backward trajectory analysis was made for altitudes of 100, 500, and 1000 m respectively to get the better results.

## Results and discussion

### Air-mass back trajectories (AMBTs) and aerosol optical depth (AOD)

**Fig. 3:**
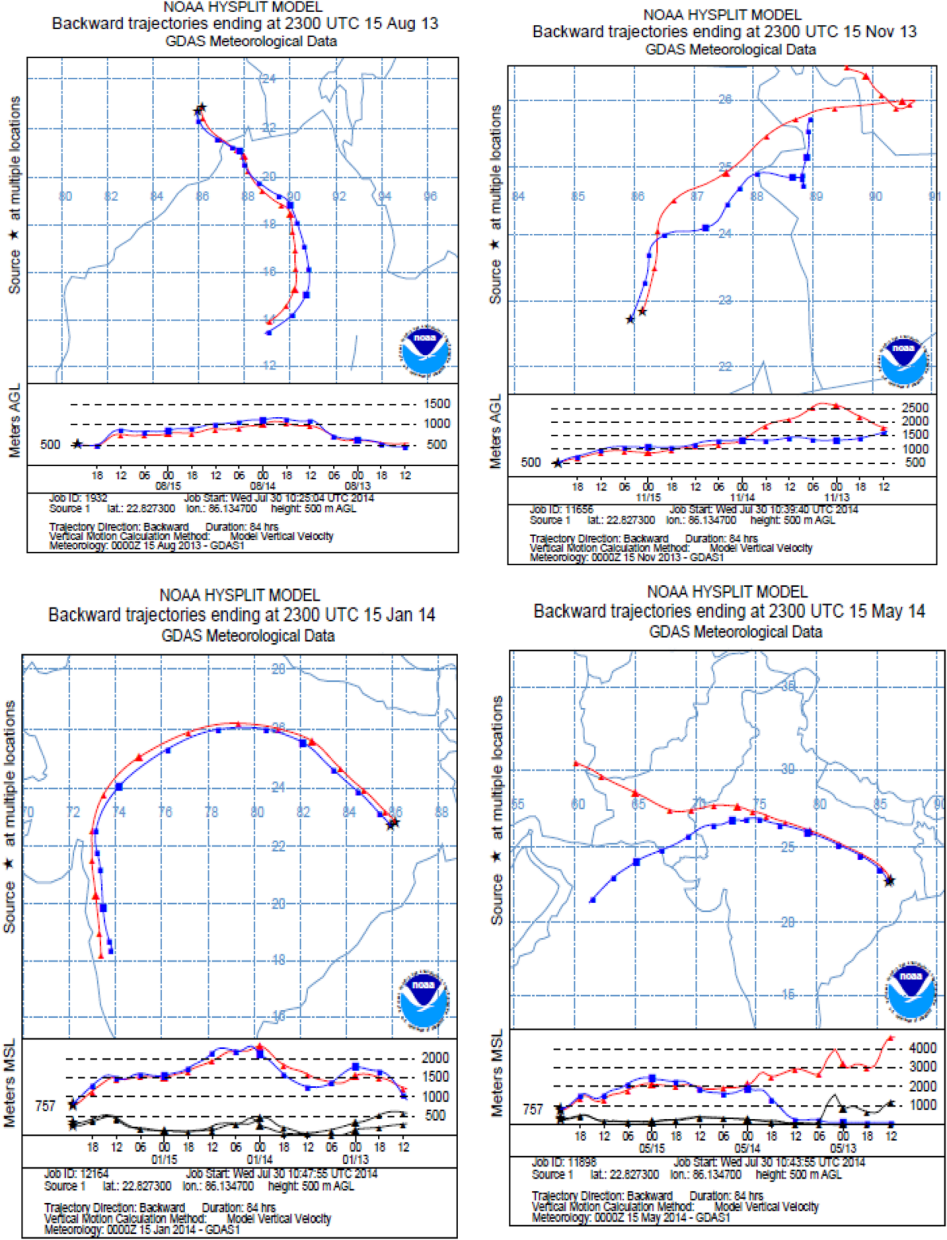
Four days’ air mass back trajectories computed during sampling season

Trajectory analyses are generally simulated in air quality studies to observe the source regions of air parcel blows into a particular region. To track the actual movement of air parcels, it is highly useful. It is also significant to consider thermodynamic factors that could stimulus the deteriorate in air quality. The Climate change will affect air quality through numerous pathways including manufacture of aeroallergens such as pollen and mold spores and increases in regional ambient concentrations of ozone, fine particles, and dust. According to Pope and Kalkstein (1996) the air mass characteristics have been successfully used over the past few decades to examine pollution concentrations, mostly with respect to diurnal air quality variability and often in the perspective of air pollutant influences on human mortality. Back trajectories were generally computed using Hybrid Single Particle Lagrangian Integrated Trajectory model (HYSPLIT-version 4; GDAS data set) from NOAA air resources laboratory (Draxler 2002). In our present study we calculated the AMBT cluster at the height of 500–4000 m (Fig. 3). The results showed that the east to west transport highly affects the chemical concentration of aerosols at Adityapur and Seraikela Kharsawan. The trajectories computed for the sampling days (15 August 2013 and 15 November 2013) clearly show the continental impact on BoB. In the present environment, sampling site is perfect to study the impact of upwind sources of the both sites in chemical composition of aerosols over the BoB. However, between 15 January 2014 and 15 May 2014, air parcel originated and transferred to western regions of the sampling site.

**Fig. 4:**
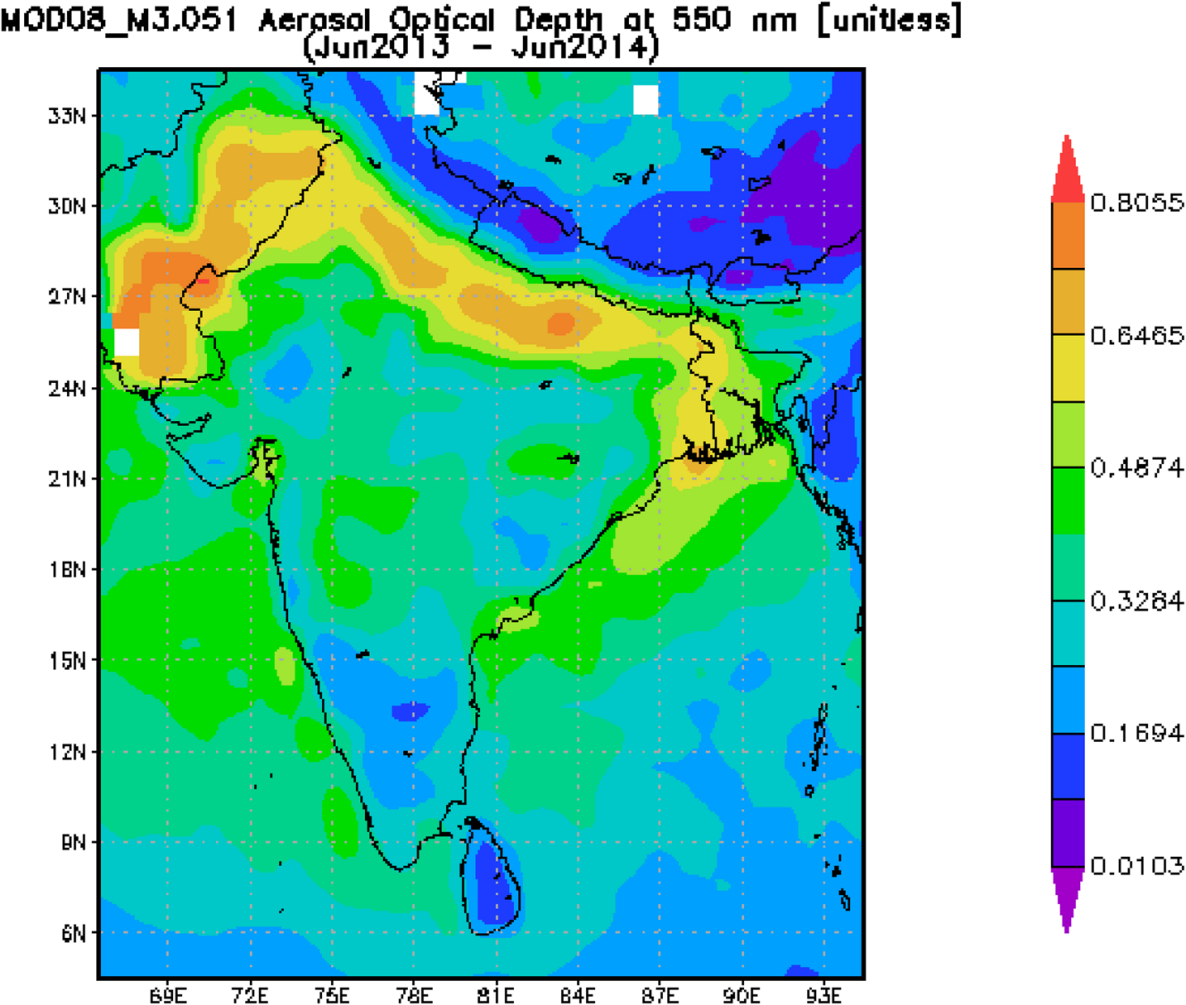
Seraikela-Kharsawan and Adityapur located in the Chhotanagpur plateau with highlighting MODIS aerosol optical depth at 550 nm over the Chhotanagpur pleatu

We have also analyzed a year (2013–2014) dataset of AOD_550_ from the MODIS sensor on board the NASA GES DISC-Terra satellite in order to evaluate the seasonal variability of specific aerosol types over the eastern India. The AOD data, representative of columnar aerosol loading, over Adityapur and Seraikela Kharsawan during sampling days. Analyses and conceptions of the data are formed by the Giovanni online data system, which were developed and maintained by the NASA. The uncertainty in the calculation of AOD for cloud free environment is less than ±0.01 for wavelength greater than 550 nm and for shorter wavelengths it is less than ±0.02, for retrieval of cloud water vapor it is ±10 %, and is less than ±5 % of the sky radiance measurements. The high load occurred mainly in the winter season, directly related to the intensity of anthropogenic emissions from industry and the burning of biomass, stagnant air masses, poor dilution of aerosols and also related strongly to the air mass movements shown by backward trajectory. Figure 4 illustrated the high MODIS AOD values at 550 nm over the study region. Computed high AOD values over the sampling site (Adityapur and Seraikela Kharsawan) strengthen the argument. The columnar AOD properties, therefore represent the resultant mixture of different aerosol types and show the seasonal changes in their nature associated with the synoptic meteorology consequently impact the radiative forcing. The eastern India constitutes an excellent atmospheric laboratory for examining the optical and microphysical aerosol characteristics, since environment is affected by locally produced anthropogenic aerosols and naturally produced particulate which is being transported to the long distances before reaching the site. Beside this, the seasonally changing air masses and the meteorological parameters also strongly affect the aerosol load and properties.

### Concentration of PM_10_

**Fig. 5:**
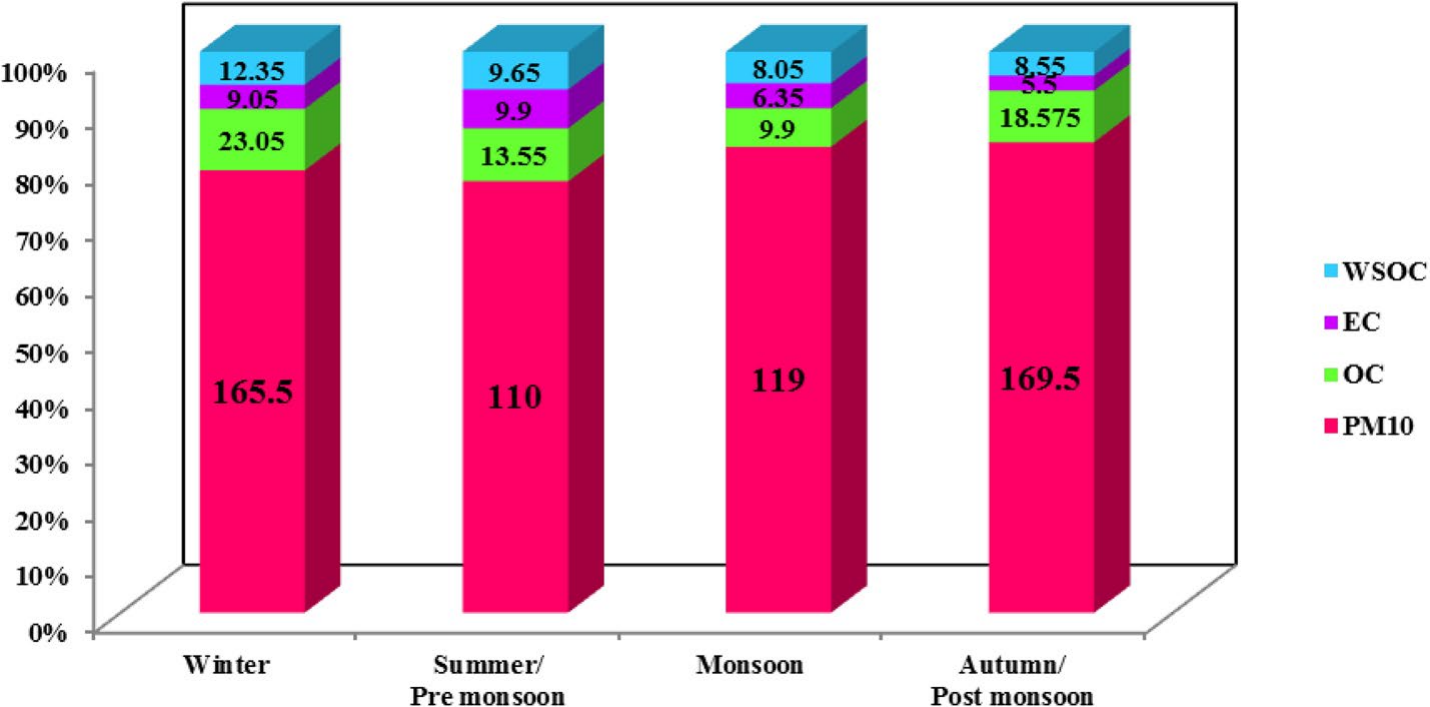
Seasonal distribution of the Adityapur campaign with their seasonal mean PM_10_ conc. of EC, OC, WSOC (µg m^−3^) for the period 1 July 2013–30 June 2014

**Fig. 6:**
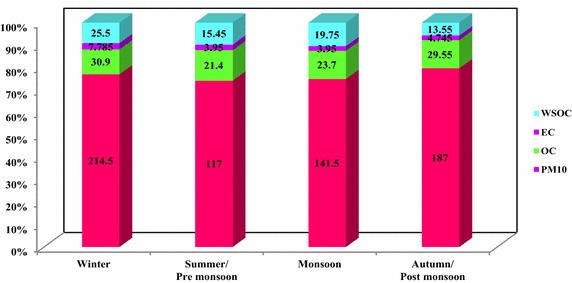
Seasonal distribution of the Seraikela Kharsawan campaign with their seasonal mean PM_10_ conc. of EC, OC, WSOC (µg m^−3^) for the period 1 July 2013–30 June 2014

The annual average mass concentration of PM_10_ over Adityapur and Seraikela Kharsawan were 165 ± 43.93 and 141 ± 30.86 varying from 76 to 275 and 64 to 244 μg/m^3^ respectively. There is a significant seasonal variation observed at both sites are presented in Figs. 5 and 6. The annual average mass value of both studies is reported higher, compared with the National Ambient Air Quality Standards (NAQS 2009) given by Central Pollution Control Board India (CPCB 2009), and also meet higher than World Health Organization standards (WHO 2005). From Figs. 5 and 6 it can be clearly seen that the Adityapur had the highest concentrations of PM_10_ in the winter (214.50 ± 65 μg/m^3^) and lowest in the summer (117 ± 40 μg/m^3^), and for Seraikela Kharsawan the highest concentrations of PM_10_ were also found in winter season (165.50 ± 51 μg/m^3^) and lowest in the summer season (110 ± 21 μg/m^3^). During the summer season the concentration of PM_10_ were lower it may be due to, the highest wind speed in local language we call as “aandhi” and due to this the pollutants disperse quickly into the atmosphere while high temperature of the atmosphere also favour. As while in the summer due to frequent dust aandhi and high temperature it is expected to be more intense photochemistry to generate secondary organics but due to dispersion of air in the summer the SOA formation is less than winter. There is on the other hand, in winter season, due to lower ambient temperatures, lower mixing depths, temperature inversion condition, low, calm condition and higher consumption of fuel augments the pollution (Ambade 2012). It can be noted that overall higher concentrations of PM at this site may be mainly due to industrial, vehicular activities, biomass and fossil fuels burning. (Hsieh et al. 2012). In addition to this during winter season, very frequent and persistent temperature inversion and foggy conditions at ground level cause a substantial quantity of aerosols to gather in the lower levels of the atmosphere. Aerosol concentrations during winter season were also largely due to massive industrial and biomass burning over eastern part of India especially in Jharkhand (Ambade 2012).

### Mass concentrations of carbonaceous species (OC, EC, and WSOC)

EC and OC in aerosol illustrate special notice in current scenario because of its unique role in the greenhouse gas and warm the atmosphere; it extremely effects on human health and climate. Both EC and OC originate as a result of incomplete combustion of motor vehicle fuel, industries, biomass and fuels used for housing cooking. (Ambade 2012).

In Figs. 5 and 6 it can be seen that OC and EC exhibited similar pattern variation in both Adityapur and Seraikela Kharsawan respectively. Seasonal concentrations of OC ranged from 4.50 to 42.40 µgm^−3^ in Adityapur and from 1.60 to 32.50 µg/m^3^ in Seraikela Kharsawan, while those of EC varied from 2.20 to 8.90 µg/m^3^ in Adityapur and from 1.30 to 11.60 µg/m^3^ in Seraikela Kharsawan (Tables 3, 4) with annual average mass concentration of OC and EC over Adityapur and Seraikela Kharsawan were 26.39 ± 4.56, 5.11 ± 1.82 and 16.27 ± 5.75 and 7.70 ± 2.10 μg/m^3^ respectively.

**Table 3:** Mass concentrations (in μg m^−3^) of OC, EC, and WSOC along with OC/EC and WSOC/OC Ratios at Adityapur

	Winter		Summer/pre monsoon		Monsoon		Autumn/post monsoon	
	Min	Max	Min	Max	Min	Max	Min	Max
Mass of PM_10_	154	275	76	158	87	196	110	264
OC	19.4	42.4	4.5	32.3	6.4	35.0	17.6	41.5
EC	6.67	8.9	2.2	5.7	3.4	4.5	2.6	6.89
WSOC	16.6	34.4	2.6	21.3	3.5	32.0	7.5	19.6
OC/EC	2.91	4.76	4.77	5.76	3.65	7.78	6.67	6.02
WSOC/OC	0.86	0.81	0.91	0.66	0.60	0.91	0.43	0.47

**Table 4:** Mass concentrations (in μg m^−3^) of OC, EC, and WSOC along with OC/EC and WSOC/OC ratios, at Seraikela Kharsawan

	Winter		Summer/pre monsoon		Monsoon		Autumn/post monsoon	
	Min	Max	Min	Max	Min	Max	Min	Max
Mass of PM_10_	120	211	77	143	64	174	95	244
OC	13.6	32.2	5.5	21.6	4.6	15.7	4.6	32.5
EC	3.5	11.6	2.5	9.3	1.3	8.1	2.4	8.6
WSOC	9.1	15.6	4.5	14.8	3.9	12.2	2.6	14.5
OC/EC	2.23	3.89	1.17	1.33	1.41	2.56	1.92	3.78
WSOC/OC	0.67	0.48	0.82	0.69	0.95	0.78	0.57	0.45

The range summaries of water-soluble organic in the tropical Indian aerosol (PM_10_) samples from Adityapur and Seraikela Kharsawan were given in Tables 3 and 4. Their temporal variations are also shown in Figs. 5 and 6.

In general, mean mass concentrations of WSOC, OC, and EC are higher during winter and significantly lower in summer and monsoon season (Figs. 5, 6). During winter, OC and EC concentrations are nearly 2–3 times lower than those during summer and monsoon season. This organized, reduce in concentrations of carbonaceous species is attributed to the altering source strength of emissions from industrial and biomass burning visa- via fossil-fuel burning and boundary layer dynamics (Ram et al. 2010b).

OC and EC mass experienced more or less significant seasonal variation in both Adityapur and Seraikela Kharsawan respectively. EC is emitted from biomass and/or fossil fuel, incomplete combustion processes as fine particles (Salma et al. 2004). The abundant carbonaceous aerosols in Saraikela Kharsawan are largely caused by the regional winter time coal-burning emission due to house heating. This is also mainly due to the far distance from EC sources (e.g. traffic and industrial emissions). Higher concentrations of EC imply that contributions from anthropogenic sources are higher in winter than in summer season. When the concentrations of OC are comparing between summer, monsoon and winter, it may be concluded that the organic aerosols have an additional sources for ex. Biogenic emissions in summer because the production of secondary aerosol is equally significant in both the seasons due to high existing solar radiations, and temperature over the region, which are adequate to endorse a photochemical process and due to elevated humidity in the monsoon season the OC and WSOC concentration has been recorded higher (Youn et al. 2013).

Coal is used in both household cooking and industrial coal burning boilers in major Indian cities including Adityapur and Seraikela Kharsawan. Both of these sources emitted large amount of carbonaceous particles reported by Zhang et al. (2000). Adityapur is a major industrial Centre of East India. It houses companies like Tata Steel, Tata Motors, Tata Power, Lafarge Cement, Telcon, TCE, TCS, Timken BOC Gases, TRF, Tinplate, Praxair and many more industries used coal while in Saraikela Kharsawan consumed major its coal in winter due to residential heating (about 16 % of coal was used for this purpose alone). In addition, it was popular to burn paddy and wheat residue in situ especially in the Seraikela Kharsawan area which is mainly surrounded by paddy field and agricultural land. For this kind of biomass burning it was reported that over 60 % was emitted as carbonaceous particulate (Watson and Chow 2001).

Studies about both industrial and remote locations have reported that WSOC accounts for approximately 20–67 % of the total particulate carbon in the atmosphere (Sempere and Kawamura 1994). The WSOC fraction in OC also shows a clear spatial variation. The Saraikela Kharsawan site has the lowest WSOC fraction, whereas the industrial site Adityapur has the highest WSOC fraction in any a given season. This may be explained by the relative contribution of primary and secondary organic aerosols. At the Adityapur, the aerosol loadings are heavily influenced by the industrial, local vehicular emission sources and other primary emission sources (e.g. cooking fumes). The Saraikela Kharsawan site has no major local emission sources, but burning of agricultural wastes and cooking fumes is the major.

### OC/EC and WSOC/OC ratios and sources of carbonaceous species

**Fig. 7:**
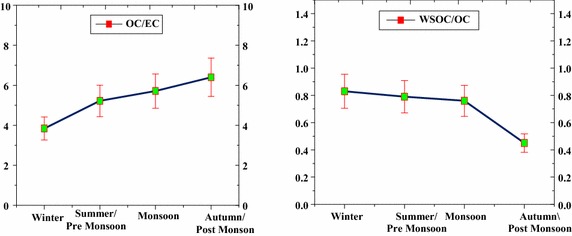
Seasonal distribution of the Adityapur campaign with their seasonal mean conc. ratio of OC/EC, WSOC/OC for the period 1 July 2013–30 June 2014

**Fig. 8:**
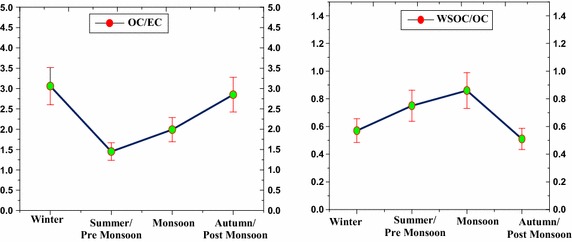
Seasonal distribution of the Seraikela Kharsawan campaign with their seasonal mean conc. ratio of OC/EC, WSOC/OC for the period 1 July 2013–30 June 2014

The mass ratios of OC to EC (OC/EC and WSOC to OC (WSOC/OC) are shown in Figs. 7 and 8. The OC/EC can be used to interpret the emission and transmission characteristics of carbonaceous aerosol. Annually OC/EC ranged from 2.91 to 7.78 with an annual average of 5.29 ± 1.08 in Adityapur, and from 1.17 to 3.89 with an annual average of 2.34 ± 0.75 in Saraikela Kharsawan. No seasonal trends are found for the OC/EC ratios in both sites. As mentioned below OC/EC ratios are quite different for various sources, of which the emissions are more or less seasonally different throughout the year. The characteristically different OC/EC ratios can be attributed to the predominance of biomass burning sources like poor combustion efficiency during wood-fuel, agricultural waste burning and Coal burning). The OC/EC and WSOC/OC ratios, along with OC and EC abundances, over Adityapur and Saraikela Kharsawan have been summarized in Table 5. Biomass burning emissions (from poor combustion efficiency during wood-fuel, farm waste burning and fossil fuel burning) have been referred as a major source of carbonaceous aerosols in India (Gustafsson et al. 2009), while coal-based emissions are highly observed in a regional part of eastern India (Reddy and Venkataraman 2002).

**Table 5:** The comparison of the OC/EC and WSOC/OC of Adityapur and Seraikela Kharsawan with previous studies

Location	Sampling time	OC/EC	WSOC/OC	References
Adityapur	July 13–June 14	5.29 ± 1.08	0.71 ± 0.17	Present study
Seraikela Kharsawan	July 13–June 14	2.34 ± 0.75	0.67 ± 0.16	Present study
Ahmadabad (urban)	Dec 07	6.2 ± 0.8	0.41 ± 0.06	Rengarajan et al. (2011)
Arabian Sea	April–May 06	~2.7	n.a	Kumar et al. (2008a)
Arabian Sea	Dec 07	4.7 ± 1.6	~0.9	Kumar et al. (2012)
Biofuel	–	~6.7	n.a	Andreae and Merlet (2001)
BoB (IGP-2006)	Mar–April 06	4.3 ± 2.4	n.a	Kumar et al. (2008b)
BoB (IGP-2009)	Jan 09	2.9 ± 0.8	n.a	Srinivas and Sarin (2014)
BoB (SEA-2009)	Jan 09	2.0 ± 1.3	n.a	Srinivas and Sarin (2014)
Hisar (IGP)	Dec 04	8.5 ± 2.2	0.64 ± 0.14	Rengarajan et al. (2007)
INDOEX-total	Jan–Mar 99	~1.3	n.a	Mayol-Bracero et al. (2002)
Kanpur (IGP)	Jan–Feb 07	8.7 ± 3.9	0.37 ± 0.09	Ram et al. (2010b)
Kharagpur	Nov 09–Mar 10	7.0 ± 2.2	0.52 ± 0.16	Srinivas and Sarin (2014)
Manora Peak	Dec 04	6.0 ± 1.9	0.78 ± 0.13	Rengarajan et al. (2007)
Manora Peak Winter	Jan 06	6.3 ± 2.2	~0.57	Ram et al. (2008)
Mt. Abu	Dec–Mar 05	4.5 ± 0.9	n.a	Ram et al. (2010a)
NCO-P	Nov 07–Feb 08	9.6	~0.66	Decesari et al. (2010)
Patial (IGP)	April–May	3.0 ± 0.4	0.60 ± 0.03	Rajput et al. (2014)
Patiala (IGP)	Oct–Nov	11 ± 2	0.52 ± 0.02	Rajput et al. (2014)
SCAR-B, Brazil, BB	2003	~8.3	n.a	Ferek et al. (1998)
Sonnblic Austria	July–sept	6.0	n.a	Legrand and puxbaum (2007)
Tanzania, Africa, BB	July–Aug 11	~6.0	~0.67	Mkoma et al. (2013)
Tanzania, Africa, BB	May–June 11	~7.8	~0.72	Mkoma et al. (2013)

*n.a* not available

The relative amount of EC and OC in ambient aerosol is very essential in deciding their overall radiative effect, because EC is a strong absorber of light but OC is by scattering in nature and can also increase cloud albedo by substituted as (cloud condensation nuclei) CCN (Gelencsér 2004). This is best evaluated by the OC/EC ratio; less the value, the higher the absorption efficiency of the carbonaceous aerosol (Novakov et al. 2000).

The WSOC/OC ratios ranged from 0.43 to 0.91 with an average of 0.71 ± 0.17 in Adityapur and from 0.45 to 0.95 with an average of 0.67 ± 0.16 in Saraikela Kharsawan. WSOC/OC ratios for vehicular emissions are usually low compared to those from industrial and biomass burning emissions. The low solubility of organic constituents from combustion of liquid fuels (diesel, petrol etc.) in water is the chief reason for lower WSOC/OC ratios. Cheung et al. (2009) reported that WSOC/OC ratios vary from 0.06 to 0.19 in the diesel particles emitted from light-duty vehicles. Previous studies that Saarikoski et al. (2008) have reported a value of 0.27 for vehicle emissions over an urban environment in Helsinki (Europe). As expected, higher OC/EC and WSOC/OC ratios were found in summer than in winter (Tables 3, 4). The higher WSOC/OC slope in the summer than in winter suggests that secondary organic aerosol formation processes produce significant amounts of WSOC during the summer. Information on the WSOC partitioning between its primary and secondary fraction can be derived by means of the EC tracer method.

The average and annual concentration of OC/EC and WSOC/OC in Seraikela Kharsawan are 5.29 ± 1.08, 0.71 ± 0.17 and 2.34 ± 0.75, 0.67 ± 0.16 respectively. The above report values of OC/EC and WSOC are higher.

### Comparison of OC/EC and WSOC/OC in Adityapur and Seraikela Kharsawan with previous studies

Table 5 summarises the comparison of the concentration ratio of EC and OC (OC/EC) and mass concentration ratios of WSOC to OC (WSOC/EC) in Adityapur and Seraikela Kharsawan with different locations in the world including the Arabian Sea and Bay of Bengal (BoB). The annual average concentration of OC/EC (5.29 ± 1.08) in Adityapur and the average concentration of WSOC/EC (2.34 ± 0.75) in Saraikela Kharsawan is higher than those reported in summer in the Arabian Sea, INDOEX-total and over the BoB but lower than that reported in Ahmadabad (urban), Hisar (IGP), Kanpur (IGP), Kharagpur, Manora Peak, Patiala (IGP), BoB, Sonnblic Austria and summer and winter of Tanzania, Africa BB (Table 5).

The major sources of carbonaceous aerosols in Ahmadabad (urban), Manora Peak, Hisar (IGP), Kanpur (IGP) and Manora Peak Winter, are expected to be biofuel combustion (cow-dung cake, wood and agricultural waste) and biomass burning (Ram et al. 2008, 2010a). Similarly, the major contributions of carbonaceous aerosols in Kharagpur, winter and summer in Tanzania, Africa, BB as well as over the BoB were attributed to be biomass burning (Mkoma et al. 2013). A Similar pattern is also reported in Adityapur and Seraikela Kharsawan.

### Formation of secondary organic carbon

Castro et al. (1999) noted that the utilize of the minimum OC/EC ratio in the ambient aerosol to be as primary origin and, thus, calculated the secondary organic carbon, OCsc. It is understood in such an approach that the minimum OC/EC ratio at the sampling location were uttered by the local meteorological conditions like, high wind speed or lack of direct solar radiation etc. that do not support the formation of secondary OC.

Castro et al. (1999) had well reported on (OC/EC) ratios in the range of 1.10 to 1.50 for the rural and urban European sites. Using the following ms equations (Castro et al. 1999), OCsc can be semi-quantitatively estimated for a definite region of concerned:

$$\begin{aligned} & {\text{OCpr}} = {\text{EC}} \times \left( {\text{OC/OCpr}} \right) \\ & {\text{OCsc}} - {\text{OCms}}{-}{\text{OCpr}} \\ \end{aligned}$$

where, OCpr the primary OC and (OC/EC)pr the primary OC/EC ratio observed during the sampling period, OCms is the measured OC in ambient aerosol and OCsc the secondary OC. On the basis of 1 year sampling at Adityapur and Seraikela Kharsawan, the (OC/EC)pr ratio were 5.29 ± 0.29 and 2.34 ± 0.75 respectively. The annual average was determined by taking the average of summer, winter, monsoon and high monsoon season OC/EC ratios, Using this ratio and assuming consistent sources, prevailing for primary OC and EC in the industrial and semi urban atmosphere, we have estimated OCsc for the sampling site at Adityapur and Seraikela Kharsawan. The choice of (OC/EC)pr ratio could be rather arbitrary with some degree of uncertainty [distant from amount of uncertainty (1σ) of ~5.7 %] arising due to the seasonal disparities in the source vigour and meteorology as well as the chemical mechanism concerning the manufacture of OCsc from oxidation products of precursors (Pandis et al. 1992; Castro et al. 1999). Hence the OCsc estimates can be considered as a lower limit of secondary aerosol formation. The estimated OCsc is found range from 8 to 35.9 % of the total OC. The temporal variation of OCsc follows the same trend as that of the WSOC which indicates a significant fraction of OCsc is water soluble. An earlier studies have exposed that WSOC can be used as a marker of OCsec in contaminated urban environment (Weber et al. 2007). Rengarajan et al. (2011) have described that the OCsec formation is seriously inclined by the aerosol acidity during winter. Tables 3 and 4 are clearly presented the value observed of OC/EC and WSOC/OC which clearly focus that the sources associated with industrial and biomass burning, primary and/or secondary, is also significant.

## Conclusions

We report first effort to data set on ambient aerosol, EC, OC, and WSOC, EC/OC and WSOC/OC in the industrial and semi urban site of Adityapur and Seraikela Kharsawan, eastern India. During study period, the annual mean mass concentration of PM_10_, EC, OC, WSOC, EC/OC, WSOC/OC of Adityapur and Seraikela Kharsawan was 165 ± 43.93, 26.39 ± 4.56, 5.11 ± 1.82, 18.56 ± 5.3, 5.29 ± 1.08, 0.71 ± 0.17 and 141 ± 30.86, 16.27 ± 5.75, 7.70 ± 2.1, 9.65 ± 1.92, 2.34 ± 0.75, 0.67 ± 0.16 respectively. Relatively high mass concentrations at Adityapur site may be attributed to industrial, biomass burning and vehicular emissions. A more meticulous attribution of carbonaceous aerosol over the eastern India to specific sources must await the result of more studies.
